# Neural connectivity abnormalities in autism: Insights from the Tuberous Sclerosis model

**DOI:** 10.1186/1741-7015-11-55

**Published:** 2013-02-27

**Authors:** Charlotte Tye, Patrick Bolton

**Affiliations:** 1 MRC Social, Genetic and Developmental Psychiatry Centre, Institute of Psychiatry, King's College London, De Crespigny Park Road, Denmark Hill, London, SE5 8AF, UK; 2 Child & Adolescent Psychiatry, Institute of Psychiatry, King's College London, De Crespigny Park Road, Denmark Hill, London, SE5 8AF, UK

**Keywords:** ASD, comorbidity, connectivity, electroencephalography (EEG), graph theory, Tuberous Sclerosis Complex (TSC)

## Abstract

Autism Spectrum Disorder (ASD) is a behavioral syndrome caused by complex genetic and non-genetic risk factors. It has been proposed that these risk factors lead to alterations in the development and 'wiring' of brain circuits and hence, the emergence of ASD. Although several lines of research lend support to this theory, etiological and clinical heterogeneity, methodological issues and inconsistent findings have led to significant doubts. One of the best established, albeit rare, causes of ASD is the genetic condition Tuberous Sclerosis Complex (TSC), where 40% of individuals develop ASD. A recent study by Peters and Taquet *et al. *analyzed electroencephalography (EEG) data using graph theory to model neural 'connectivity' in individuals with TSC with and without ASD and cases with 'idiopathic' ASD. TSC cases exhibited global under-connectivity and abnormal network topology, whereas individuals with TSC + ASD demonstrated similar connectivity patterns to those seen in individuals with idiopathic ASD: decreased long- over short-range connectivity. The similarity in connectivity abnormalities in TSC + ASD and ASD suggest a common final pathway and provide further support for 'mis-wired' neural circuitry in ASD. The origins of the connectivity changes, and their role in mediating between the neural and the cognitive/behavioral manifestations, will require further study.

Please see related research article here http://www.biomedcentral.com/1741-7015/11/54

## Background

The recent and rapid rise in the prevalence of Autism Spectrum Disorder (ASD) has fuelled intensive research into the causes and neurobiological basis of ASD, with major advances in identifying the genetic factors involved [1]. Nevertheless, the findings have revealed considerable etiological heterogeneity and marked variability in phenotypic expression, which has led to uncertainty as to whether there exist several different subtypes of ASD or whether the causal factors converge in a final common pathway. Accordingly, molecular genetic research has begun not just to study the genetic variants associated with the clinical phenotype of ASD, but also the association with neurobiological processes that may underlie the complex behavioral profile. The challenge of disentangling the heterogeneity of ASD has also led to an increasing realization that important insights can be gained from the study of the established genetic causes of ASD, where the pathophysiology is better understood and more tractable to study [2]. Importantly, in most of the genetic syndromes associated with ASD, only a subset of individuals actually develop ASD, providing a valuable opportunity to investigate the causal pathways leading to the variation in outcome. The dramatic advances in understanding the molecular mechanisms underlying fragile × syndrome and Tuberous Sclerosis Complex (TSC; two of the best established causes of ASD [3]) and the concomitant and exciting advances in drug therapies for these conditions, have highlighted the importance of these model systems for understanding whether and how ASD develops and if, as pre-clinical studies suggest, cognitive and behavioral outcomes are improved by the novel therapies [4]. At present, the etiological mechanisms underlying the co-occurrence of ASD in genetic syndromes, and the extent and depth of 'homology' in phenotypic manifestations of ASD in genetic syndromes compared with idiopathic cases of ASD, remain unclear.

In this context, the report by Peters and Taquet *et al. *[5] in *BMC Medicine *holds special interest. The study exploits recent advances in brain science to investigate the structure and integrity of neural connections and networks: the results are striking in showing that the perturbations in neural connectivity observed in individuals with TSC and co-occurring ASD parallel those observed in cases of idiopathic ASD, indicating that there is some 'homology' in the nature of the connectivity abnormalities and pointing to common pathophysiological processes.

### The mis-wired brain

In parallel with the increasing awareness that the brain operates in a complex and dynamic manner, evidence is accumulating to suggest that altered neural connectivity, rather than a specific and localized deficit, underlies many complex psychiatric disorders [6]. The advent of methodological and analytical advances has allowed the examination of brain connectivity *in vivo*. Their application to brain data in ASD has led to the suggestion that it is a developmental disconnection syndrome [7], characterized by 'local over-connectivity and long-distance under-connectivity' [8]. Graph theory provides a quantitative framework to examine connections of the brain defined by a collection of brain regions (nodes) and the connections between them (edges) (see Peters and Taquet *et al. *[5] and [9]). Aberrant connectivity across different modalities is widely reported in ASD: findings support reduced functional connectivity measured using magnetic resonance imaging (MRI), reduced fractional anisotropy (FA) as an index of structural connectivity using diffusion tensor imaging (DTI), and altered yet inconsistent connectivity findings using electroencephalography/magnetoencephalography (EEG/MEG; see [10] for comprehensive review). Peters and Taquet *et al. *[5] show a decreased ratio of long- over short-range coherence in ASD, suggesting that local brain regions communicate via strong connections compared to long-range paths, although with no significant differences in functional graph theoretical measures.

Previous results differ with respect to the regions in which aberrant connectivity has been observed, age of the participants and connectivity patterns during resting state versus task-dependent paradigms. Moreover, the differences in findings may also reflect the heterogeneity of ASD. Accordingly, a focus on a simpler model of ASD, such as TSC, holds the promise of being especially informative. In contrast to ASD, Peters and Taquet *et al. *[5] report a more widespread network dysfunction in TSC, with decreased global efficiency and local connectivity and, as a result, closer to the classic definition of a 'disconnection syndrome'. Findings suggest, therefore, that abnormal connectivity patterns are associated with these disorders, yet the basis for these abnormalities is unknown, for example, whether they involve the whole brain or specific brain regions. In addition, whether or not these findings depend on the frequency band studied, given the varied associations between frequency and cognition is of interest. The Peters and Taquet *et al. *[5] study focused on alpha and theta bands, and it would be useful to investigate gamma activity considering its hypothesized role in neural synchronization [11].

### Targeting the common pathway

While it appears likely that in most cases there are multiple risk pathways to ASD, it is possible that these pathways converge at some level, with network efficiency providing a potential platform. As such, system-level concepts may simplify and merge the diverse genetic mechanisms identified for ASD. One hypothesis is a greater ratio of excitatory versus inhibitory connections in the brain networks in ASD, supported by hyper-connectivity in frontal brain regions, elevated gamma frequency and an increased risk of seizures [12]. In addition, many autism susceptibility genes are linked to activity-dependent neural responses, supporting the emerging claim that ASD is a disorder of the synapse [13]. A recent hypothesis for the emergence of ASD in TSC and related conditions is dysregulation of neurotrophic factors, including brain-derived neurotrophic factor (BDNF) which plays a role in protein regulation through inhibition of TSC1-TSC2 signaling and mutations in *PTEN *that lead to excessive mammalian Target of Rapamycin (mTOR) signaling. It is suggested that these processes alter the development of inhibitory synapses and synaptic plasticity, ultimately resulting in syndromic ASD [13].

An important finding from Peters and Taquet *et al. *[5] is that despite methodological limitations, the connectivity abnormalities associated with ASD were confined to the subset of TSC + ASD cases, which suggests that there may be distinct risk factors for ASD in TSC. This highlights the need to investigate the interplay between risk factors [14,15]. Beyond the molecular and synaptic level, the research has also to account for the raised risk of ASD following infantile spasms (a form of epilepsy) in cases of TSC. While these observations provide insight into the mechanisms underlying autism in the context of TSC, being able to identify the common pathway would also facilitate improved treatment across a range of different causes of ASD, and provide a model for other Mendelian disorders [16]. It is important not to oversimplify, however, and to recognize that targeting dysregulation in one pathway may potentially worsen outcomes in cases that are due to abnormalities in other paths, as has recently been shown in animal models of TSC and fragile × syndrome [17].

### A developmental perspective

An important outstanding question is whether the connectivity abnormalities reflect earlier developmental and compensatory processes. As such, studies of the emergence, maturation and developmental sequences in connectivity and associated network properties are required, especially as there are such vast age-related changes in EEG. In particular, it is unclear whether the altered connectivity patterns occur pre- and/or post-natally and reflect deterioration of already established connections or a failure in the formation of connections. Some findings suggest over-connectivity at a young age and under-connectivity at older ages in ASD, which may reflect impaired synaptic pruning (and therefore overgrowth) during brain development [10]. For example, by occupying sites normally available, the exuberance of axons over shorter distances may restrict longer distance connections, which develop later and need additional time to grow [18]. As such, the increased resilience to 'targeted attack' in ASD reported in Peters and Taquet *et al. *[5] may reflect this redundant over-connectivity or reduced functional specialization at younger ages. Accordingly, treatments targeting dysfunction in excitatory systems may be effective in early development, whereas later it may be necessary to also treat the ensuing alterations to neural networks. Quantitative measures, such as those derived from graph theory, may therefore be used as biomarkers for early detection, to inform prognosis, and to allow the formation of more homogenous groups based on emergence and persistence of key behavior and brain markers [19].

## Conclusions

The study by Peters and Taquet *et al. *[5] exemplifies the insights that can be gleaned from studying rare genetic disorders to elucidate etiological pathways in complex psychiatric disease, as well as the use of state-of-the-art neuroscience techniques to map abnormalities in brain function. The findings are advancing our understanding of the neurobiology of ASD. More research of this kind is needed, and the results of their prospective studies will be eagerly awaited.

## Abbreviations

ASD: autism spectrum disorder; BDNF: brain derived neurotrophic factor; DTI: diffusion tensor imaging; EEG: electroencephalography; FA: fractional anisotropy; MEG: magnetoencephalography; MRI: magnetic resonance imaging; mTOR: mammalian Target of Rapamycin; TSC: Tuberous Sclerosis Complex.

## Competing interests

The authors declare that they have no competing interests.

## Authors' contributions

CT drafted the manuscript. PB made substantial contributions in revising it. CT and PB read and approved the final manuscript.

## Author's information

CT is a postdoctoral researcher at the Institute of Psychiatry, Kings College London. PB is Professor of Child and Adolescent Psychiatry at the Institute of Psychiatry and Honorary Consultant Child and Adolescent Psychiatrist at the Maudsley Hospital, where he leads the National Specialist Clinical Service for children with autism spectrum and related disorders, including TSC.

## Pre-publication history

The pre-publication history for this paper can be accessed here:

http://www.biomedcentral.com/1741-7015/11/55/prepub
